# The Impact of Job Insecurity on Organisational Citizenship Behaviour and Task Performance: Evidence from Robotised Furniture Sector Companies

**DOI:** 10.3390/ijerph18020515

**Published:** 2021-01-10

**Authors:** Živilė Stankevičiūtė, Eglė Staniškienė, Joana Ramanauskaitė

**Affiliations:** School of Economics and Business, Kaunas University of Technology, Gedimino g. 50, LT-44249 Kaunas, Lithuania; egle.staniskiene@ktu.lt (E.S.); joana.ramanauskaite@ktu.lt (J.R.)

**Keywords:** job insecurity, task performance, organisational citizenship behaviour, robots, robotisation, furniture industry

## Abstract

Over the past decade, in the light of intensive robotisation, job insecurity referring to the employees’ overall concern about the continued availability of their jobs in the future has become a hot topic. A general assumption supported by the findings is that job insecurity causes far-reaching negative consequences for the employee well-being and health, attitudes towards the job and organisation, and behaviours at work. However, the focus on behavioural outcomes, especially on employee performance at work, is still scant. Trying to narrow the gap, the paper aims at revealing the linkage between job insecurity and two dimensions of performance, namely task performance and organisational citizenship behaviour. Building on the hindrance stressor dimension of the stress model, the paper claims that a negative relationship exists between the constructs. Quantitative data were collected in a survey of robotised production lines operators working in the furniture sector in Lithuania. As predicted, the results revealed that job insecurity had a negative impact on both the task performance and organisational citizenship behaviour. These findings affirmed that job insecurity was a hindrance stressor, which needed to be considered when managing human resources in a robotised production environment.

## 1. Introduction

Work has been a subject of transformation for a couple of centuries [1,2,3]. In particular, recently these changes have been accelerated by the rapid technological advancement [4,5,6,7]. Worldwide, the companies have started adapting technologies with intention to “*decrease costs, generate additional revenues, provide consistent product quality, streamline operations, expand production/service capacity, improve company’s competitiveness*” ([8], p. 17). This is particularly valid for the manufacturing sector, where industrial robots have been used now for several decades [8,9]. Such a situation raises an important concern about technological unemployment when humans are replaced or complemented by machines [10,11]. Given this focus, employed people might feel insecure about the future of their jobs [12] and feel threated by unemployment [13]. In other words, employees experience job insecurity which is situated midway between employment and unemployment [13] and is considered as a work stressor [14].

While a growing body of prior studies have investigated the detrimental effect of job insecurity on work-related well-being [15], health [16], and employee attitudes towards the job and organisation [17,18], behavioural outcomes have been studied to a somewhat lesser extent [19,20,21,22,23]. Incidentally, this is despite the notion that behaviour outcomes, such as employee performance at work, serve as a core driver for overall organisational performance and sustainable survival [22]. In such, the current paper is designed to narrow the gap by analysing the ways the job insecurity relates to employee performance referring to its two empirically related, but conceptually distinct dimensions, namely task performance and organisational citizenship behaviour (OCB). While task performance comprises job duties relating to the organisation’s technical core, OCB encompasses varying facets of discretionary work behaviour that create a positive work context while supporting the technical core [24].

Going further, it is important to underline that scant prior research demonstrated controversial findings regarding the job insecurity and employee performance. Some of them reported a negative association between the constructs [22,25,26], while others revealed a positive linkage [22]. As such, the current paper could benefit to the research in the field of job insecurity by providing further evidence regarding the nature of impact.

Moreover, it seems that the previous research has largely neglected the relevance of samples, countries, and the nature of socio-technological changes while addressing job insecurity and its consequences [11]. As it was mentioned before, manufacturing is one of the sectors with the growing number of industrial robots [9] having the potential to replace human workforce [7]. As to the best knowledge of the authors of this paper there is a no research exploring job insecurity in furniture industry with participants of the study being robotised production line operators, the current paper serves as a good starting point for opening an avenue for further investigations.

The aim of the paper is to reveal the linkage between job insecurity and employee performance, in terms of task performance and OCB among the robotised production line operators in the furniture sector. In doing this, the paper seeks to answer the following: (a) whether and to what extent do the workers feel job insecurity? (b) whether and to what extent do the workers perform tasks and demonstrate OCB? (c) whether the ratings of job insecurity and performance differ according to demographic characteristics of respondents? (d) will job insecurity impact task performance and OCB? What are the negative or positive aspects of such impact? To answer these questions, this paper analyses data from the survey carried out in the plants of the furniture sector, which is far-advanced in using robots and automation solutions. The sample consisted of operators working on robotised production lines.

The paper intends to contribute to the literature in several ways. First, the intention is to respond to the recent call of Sverke et al. [22] to focus on and extend the empirical evidence supporting the notion that job insecurity is associated with task performance and OCB. Next, in order to understand how job insecurity is related to employee performance, the hindrance–challenge occupational stressor model [27] was used. More specifically, job insecurity was treated as a hindrance stressor, which causes impaired performance. The purpose of this is to contribute to the research stream theoretically arguing and empirically supporting the notion that job insecurity is a hindrance stressor. Finally, echoing the concerns of Nam [11], the paper addresses a specific context and sample, namely robotised production line operators in the furniture sector. The perceived job security analysed here is supposed to be forward-looking, reflecting the expectations of job changes while respondents have already been affected by automation-robotisation related solutions, as they work together with robots.

The remainder of the paper is structured as follows. The theoretical part gives an overview of the literature on changing work nature and describes job insecurity, task performance and OCB. Later, the hypotheses are developed. Then, the research method applied is described. The empirical results and discussion come further. Finally, conclusions are drawn.

## 2. Theoretical Background

### 2.1. The Changing Nature of Work (in Manufacturing Industry) Due to Robotisation

Addressing our time as an era of conscious social change, Bowen and Mangum [28] claim one of the predominant factors underlying current social changes is the advancement of technology. There are no doubts that the current wave of technological innovation, known as the “Fourth Industrial Revolution” [29], will have a far-reaching impact on the labour market [4]. Technology is believed to effect economies, employment and nature of work dramatically [30,31,32]; however, it is still controversial whether the new technologies substitute or create more and new jobs [4,33,34,35].

Turning to manufacturing industry, the use of robots is at the core of debates, as industrial robots are quite widely used in manufacturing [7]. The word ‘robot’ was first introduced in 1921 and now refers to machines that can navigate through and interact with the physical world of factories, warehouses, battlefields, and offices [36]. Playing a vital role in manufacturing industry’s efforts to be competitive, industrial robots are dominating in comparison to collaborative robots [37]. Industrial robots are fully autonomous machines that do not need a human operator and that can be programmed to perform several manual tasks such as welding, assembling, handling materials, painting, or packaging [38]. Moreover, robotic technologies are continuing to advance allowing them to perform various and increasingly difficult tasks [9] making people worry that large-scale job losses are looming [34]. There are many arguments why a company would prefer using robots rather than human employees. Here, only some of them are provided. Firstly, a robot can work 24/7, without the need to relax and recover. Next, robots can implement the same routine, tedious and/or dangerous work repeatedly, correctly and in a timely manner. Further, robots focus on work without negative emotions; they do not complain or require better working conditions. Finally, robots could be rented or bought easier than human employees get hired [8]. Relying on the advantages of robots, it is not surprising that employees may feel insecure concerning the future existence of jobs [11]. The insecurity might even be fueled by some empirical evidence, as for instance Acemoglu and Restrepo [38] found that an increased use of robots has reduced the employment-to-population ratio. Naturally, technologies do not affect all work in the same way [see Acemoglu and Autor [39] for an overview]; however, human employees working in manufacturing are in real danger, as “*those employees whose jobs include repetitive, tedious, and/or dangerous tasks, and are subject to strict algorithmisation*” ([8], p. 3) are on the road for some replacement or substitution. Drawing upon the detrimental consequences of job insecurity on employees and organisations, the question of the extent of job insecurity perceived by the manufacturing employees is highly relevant. The conceptualisation of this social phenomenon is provided below.

### 2.2. Job Insecurity

Job insecurity has been defined in various ways in the literature. One of the earliest and of most-quoted definitions was provided by Greenhalgh and Rosenblatt [40], claiming that job insecurity was “*the perceived powerlessness to maintain desired continuity in a threatened job situation*” (p. 438). Another highly-quoted definition belongs to De Witte [13] arguing that job insecurity is “*the perceived threat of job loss and the worries related to that threat*” (p. 1). The overview of definitions [12] suggests that some researchers treat job insecurity as multi-dimensional, differentiating between quantitative (threats to the continuation or loss of the job itself) and qualitative (threats to continued existence of valued job features) job insecurity [13,41]. Further, another distinction often made is between the cognitive and affective job insecurity [13,22]. Cognitive job insecurity refers to the perceived threat to the continuity of one’s employment and/or to the features of the job (e.g., deterioration of working conditions), whereas affective job insecurity captures the emotional reactions to the perceived threat to one’s job (e.g., concern, worry, anxiety) [42]. However, despite the diversity in definitions, some common characteristics could be underlined. First, job insecurity is a subjective experience implying that the same objective situation (e.g., a decline in demand for the goods the company is producing) may be interpreted in various ways by different workers. Some employees may feel insecure whereas their job continuity is (‘objectively speaking’) not in danger, while others may feel secure about their jobs, even though they will be laid off soon afterwards [13]. Second, job insecurity is a future-focused phenomenon [43]. Job insecurity reflects a forecast about a loss event, which might occur one day in the future [12]. Consequently, employees are ‘groping in the dark’ as far as their future within the organisation is concerned [13]. Third, job insecurity refers to involuntary nature; the feeling of powerlessness is also a part of many job insecurity definitions [13]. Finally, job insecurity is about the stability and continuity of one’s current employment and accordingly differs from employability, as a related construct, which captures an individual’s perceived ability to obtain a new job [13]. The current paper treats job insecurity as a global construct comprising a perceived, unwelcome threat to the current job, as suggested in a study of Sverke et al. [22].

As mentioned earlier, job insecurity might generate a vast range of outcomes for individuals and organisations. Seeing that the paper limits its scope to a single outcome–performance, the next subsection describes employee performance, revealing the nature of task performance and OCB.

### 2.3. Task Performance and OCB

Employee performance refer to “*actions, behavior and outcomes that employees engage in or bring out that are linked with and contribute to organizational goals*” ([44], p. 216). In contemporary research, employee performance is treated as a phenomenon, which consists of several distinct types, or dimensions of performance behaviour [45]. This paper limits its focus to two dimensions, namely task performance and OCB.

In work psychology literature, task performance is defined as the effectiveness with which job incumbents perform activities that contribute to the organisation’s technical core either directly by implementing a part of its technological process, or indirectly by providing it with the necessary materials or services [46,47]. Very similarly, Van Scotter [48] claims that employees are engaging in task performance when they “*use technical skills and knowledge to produce goods or services through the organisation’s core technical processes, or when they accomplish specialized tasks that support these core functions*” (pp. 80–81). Pradhan and Jena [45] argue that task performance “*comprises of job-explicit behaviours which include fundamental job responsibilities assigned as a part of job description*” (p. 71). As task performance is primarily facilitated through task knowledge, task skill, and task habits [49], the primary antecedents of it include the ability to do the job and prior experience [45]. Summing up, task performance refers to activities that contribute to the technical core and these activities are formally recognised as part of the job [50].

OCB is a quite different dimension of employee performance representing contextual performance. OCB is a kind of prosocial behaviour demonstrated by individuals in a work set-up [45]. Originally defined by Organ [51], OCB is treated as “*individual behaviour that is discretionary, not directly or explicitly recognised by the formal reward system, and in the aggregate promotes the efficient and effective functioning of the organisation*” (p. 4). Later, Organ [52] provided a more precise understanding of OCB referring to the “*performance that supports the social and psychological environment in which task performance takes place*” (p. 95). According to Bolino and Turnley [53], employee efforts that go “*above and beyond the call of duty*” (p. 60) reflect the nature of OCB. More precisely, employees are engaged in OCB, when they voluntarily help colleagues who are getting behind, act in ways that maintain good working relationships, put in extra effort to complete an assignment on time or carry out task activities that are not formally part of the job [48]. In general, OCB is highly relevant because it contributes to the overall organisational effectiveness in ways that shape the organisational, social, and psychological context that might foster the task activities [47].

Generally, both dimensions of performance, as much task performance as OCB, are significant for organisational survival leading to the demand to strengthen the employee performance. However, it seems likely that job insecurity might cause the opposite result with respect to employee performance. Thus, the next subsection provides a theoretical justification and the empirical evidence regarding the association between job insecurity and task performance and OCB.

### 2.4. Job Insecurity in Relation to Task Performance and OCB

Drawing upon previous literature, it seems that stress theory [27], psychological contract theory [54], and social identity theory [55] are the main theoretical approaches used to explain the relationship between job insecurity and performance in terms of task performance and OCB. The current paper limits its focus only to the stress theory.

Recognisable technological innovations implemented by the manufacturing companies while installing increasingly more industrial robots cause some threats for the employees and act as a main stressor to employment and job existence [11,14]. Relying on the notion that job insecurity is a stressor, the explanation of the way the employees respond to job insecurity in terms of their performance is not so straightforward. The prior literature proposed a two-dimensional work stressor framework with respect to the stressors’ relationships with performance [56]. Drawing upon this two-dimensional stressor model, any stressor reflects two basic dimensions, namely hindrance and challenge [56]. A hindrance stressor is defined as excessive or undesirable work-related demands or circumstances that interfere with or hinder an individual’s ability to achieve goals [57]. Contrary to the hindrance stressor, a challenge stressor is seen as a job demand creating the opportunity for better work achievements [27]. Thus, on one hand, as a result of the hindrance stressor or “bad” stress [56], job insecurity might lead to reduced task performance and reduced OCB. However, on the other hand, as a result of the challenge stressor or “good” stress [56], job insecurity could trigger higher task performance and OCB when employees cope with job insecurity actively by exerting extra effort to demonstrate their worth, as a form of job preservation strategy [27].

Having the context of the paper in mind, this research treats job insecurity as a hindrance stressor. Unquestionably, during the last decades, technological innovations have dramatically changed the nature of work [11,30], while some labour in terms of production of goods is performed by robots, meaning that humans will be increasingly marginalised from the production process [7]. Alongside the initial high expectations of the robots’ potential to assist humans in the manufacturing process, people have become deeply concerned about the existence of their job in the future. Actually, work has extraordinary importance for individuals, as it fulfils various fundamental human needs, for instance the need for survival [14]. In the meantime, job insecurity limits or makes it impossible to satisfy some of the fundamental needs. Moreover, humans might feel that robots are their competitors in terms of being employed in a particular industry or workplace. As the technological progress accelerates and displacement effect becomes an obvious reality with manufacturing companies using robots instead of humans [31], employees might experience a feeling of powerlessness to change the current and future situations as regards their job existence. Hence, the growing use of robots raises stressful demands, which have the nature of a hindrance stressor. In general, hindrance stressor has the potential to harm personal growth, cause negative emotions, and trigger a passive style of coping [56]. One way to emotionally cope with such a stressor is to behaviourally withdraw from the situation [19]. Turning to performance, reduced task performance and reduced OCB are the perfect examples of such behavioural withdrawal [19]. Referring to job insecurity as to an undesirable work-related demand, employees might demonstrate lower task performance and lower discretionary efforts.

Turning to empirical evidence regarding the linkage between job insecurity and task performance and OCB, no consensus exists. The majority of studies have found job insecurity to be negatively related to general and task performance [20,43,58]; however, there are some studies that have found non-significant [55] or even positive associations [59]. Regarding OCB, the associations between job insecurity and OCB have also proved to be negative in most studies [22], although some studies have found non-significant relationships [20].

Looking to meta-analysis, Sverke et al. [2] found no significant relationship between the job insecurity and performance. Subsequent meta-analyses [25,26,42] found a rather weak, but nevertheless statistically significant negative relationship. A more recent meta-analysis of Sverke et al. [22] demonstrated that job insecurity was associated with impaired task performance and OCB.

In summary, the core assumption of the current paper claims that the hindrance effect of job insecurity manifests itself through behavioural withdrawal (lower task performance and OCB), because it reflects a passive coping process. In line with this perspective, job insecurity makes it difficult for employees to devote their energy and attention to performing their duties or to demonstrating extra-role performance [27]. Based on theoretical reasoning while treating job insecurity as a hindrance stressor, the paper hypothesises the following:

**Hypothesis** **1** **(H1).** *Job insecurity will be negatively related to task performance.*

**Hypothesis** **2** **(H2).** *Job insecurity will be negatively related to OCB.*

**Figure 1:**
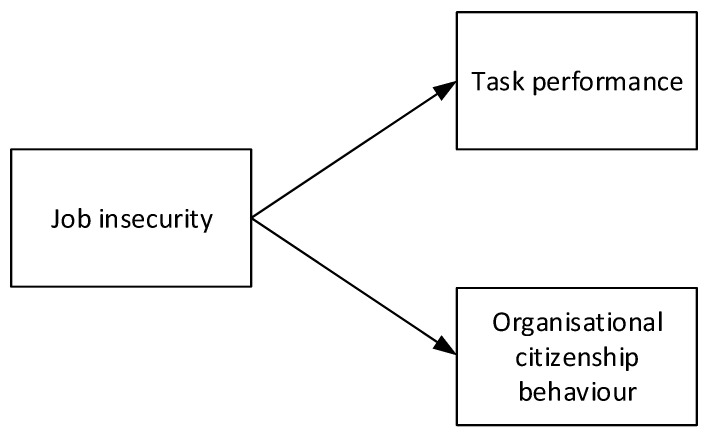
Theoretical model.

The theoretical model is provided below (Figure 1).

## 3. Methodology

### 3.1. Context

The furniture industry is one of the oldest wood processing industries in Lithuania. The Lithuanian furniture industry has a relatively cheap and qualified labour force; however, its shortage is experienced because of labour-intensive products [60]. Lukšytė and Melnikas [60] also recognise that Lithuanian furniture industry is one of the most competitive and highly developed manufacturing industries in Lithuania; it uses innovative production methods making high quality furniture; has long-standing traditions; is applying high technologies; its staff possess appropriate qualifications and competencies; it is integrated into the global economy. The latter statement can be supported by the fact that 69.4% of furniture made in Lithuania was exported to the EU market in 2019 [61]. Overall, the Lithuanian furniture industry created 2.4% of national GDP and accounted for 9.7% of GDP of the manufacturing sector, with a production value of approximately 1.4 billion euros in 2015 [62]. In 2019, the value of sales of goods and works performed in the sector increased by 20% [61]. The Lithuanian furniture industry shares around 0.1% of the market realized revenue (worldwide US$1417 bn) [63]. The main indicators of Lithuanian furniture industry are presented in Table 1. There were 872 companies operating in the furniture industry sector in Lithuania with 27,724 employees in 2015. Total employment during that year was 1,468,900 people working in 71,445 enterprises [61].

It should be noted that the development of wood industry enterprises is associated with modern technological equipment and automation of production processes [60]. Lithuanian industrial companies seem to understand the challenges they are facing—the demand for robotisation solutions currently exceeds the supply [64,65], even though only 3% of Lithuania-based enterprises employ robotic solutions currently [66].

The Lithuanian furniture industry workforce is encountering the issues of robotisation; it is estimated that because of automation more than 60% of jobs are at risk in Lithuania, with 20% of those facing high risk of significant change (more than 70% probability) [67]. 3D printers might easily carry out the production of furniture that is designed by algorithms [63]. Nevertheless, Lithuania is highly dependent on IKEA, that is both the main engine driving the innovation and progress of the Lithuanian furniture industry, making it more labour- and cost-efficient, but also posing higher risks that is brought by lack of market diversification [68]. However, the Lithuanian furniture industry is expected to double in size in the next several years because of the ongoing clusterisation [69]. Thus, concluding, the Lithuanian furniture industry is and should remain one of the most significant and highest added-value sectors for the Lithuanian economy, as well as for the labour market [60].

### 3.2. Sample and Data Collection 

The respondents chosen to gather the data and test the hypotheses were operators of robotised furniture production lines in Lithuania. Five furniture companies were surveyed in 2020. The research was based on the criterion of convenience in order to obtain the data from the respondents who were easier to reach; however, certain inclusion criteria were applied: (1) working in furniture manufacturing industry; (2) working on robotised production lines. Paper questionnaires were distributed to the employees. Data collection took approximately 1 month. At the end of the research, 350 questionnaires were collected from five furniture companies. According to the numbers of the employees working in the Lithuanian furniture sector (27,724), such an amount of responses reflected an acceptable bias of 5.4%, which indicated the reliability of the data. Concerning the respondent profile, data about gender, age, education, and working time in the organisation were collected. The socio-demographic characteristics of the sample are reported in Table 2.

As it is visible in Table 2, 61 percent of the 350 respondents were male employees. A small number (5.0%) of respondents belonged to generation Z (date of birth 2002 and later), 50.0% were representatives of generation Y (date of birth 1981–2001), 35.0% of those surveyed belonged to generation X (born in 1965–1980), and 10.0% were people from the Baby boom generation (born in 1946–1964). Regarding the education level of respondents, 51.0 percent held a higher education degree. Turning to job tenure, 4.0% of respondents had worked less than 1 year for the particular company, 21.0% of respondents had a 1–3 year working experience, 38.0% of respondents reported that they had worked for the organisation from 3 to 5 year, 30.0% of respondents declared a 5–10 year working experience, while 7.0% reported that they had worked for the particular organisation 10 years and longer.

### 3.3. Measures

Job insecurity was measured by using a four-item scale developed by De Witte [70]. Response options ranged from 1, “strongly disagree”, to 5, “strongly agree”. Sample items are: “I feel insecure about the future of my job” and “I am sure I can keep my job” (reverse coded). Cronbach’s alpha was 0.675.

Task performance was measured using a three-item scale developed by Goodman and Svyantek [71]. Response options ranged from 1, “strongly disagree”, to 5, “strongly agree”. Sample item is “I fulfil all the requirements of my job “. Cronbach’s alpha was 0.576.

OCB was measured using a five-item scale developed by Verburg et al. [72]. Response options ranged from 1, “strongly disagree”, to 5, “strongly agree”. A sample item is “I assist others with their work, even when they do not ask directly “. Cronbach’s alpha was 0.807.

All items are provided in Appendix A.

Task performance and OCB were measured via self-rating.

### 3.4. Data Analysis

The statistical package IBM SPSS Statistics Standard v.23 (IBM, Armonk, NY, USA) was used to perform statistical analysis. Respondents’ demographical data were summarised using descriptive statistics. The Spearman’s coefficient was used as a means of bivariate correlations between the main variables of the study as the data had a skewed distribution. Logistic regression analyses were conducted to explore the relations between job insecurity and task performance and organisational citizenship behaviour factors. Multiple logistic regression analyses using an enter method were conducted. Models were adjusted for personal factors (gender, age, level of education, and years worked for the organisation). Results were considered statistically significant at a 5% (*p* < 0.05) significance level. Scores for all scales used were calculated. Nonparametric statistics were applied since none of the scales were normally distributed. Median and interquartile range were selected as descriptive measures. All study groups were compared using Kruskal–Wallis tests. Mann–Whitney tests were used to analyse the scores between two groups when there were significant differences.

Because self-reported data were used, there is a need to check for potential biases resulting from common method variance (CMV). Actually, researchers can control CMV with statistical and procedural remedies [73,74]. Based on recommendations [74], some procedural remedies were used by protecting respondent anonymity, improving item wording, and separating the measurement of the predictor and outcome variables. Turning to statistical remedies, Harman’s one-factor analysis was performed as a statistical remedy. According to Harman’s one-factor analysis, CMV was not a serious issue in the data.

## 4. Results

The means, standard deviations, and correlation matrix are provided in Table 3.

Referring to Table 3, a negative correlation between job insecurity and task performance (−0.261, *p* < 0.01) was revealed. The same situation was observed in respect to OCB, seeing that a negative correlation between job insecurity and OCB (−0.249, *p* < 0.01) was found.

Previous research has suggested that employees according their demographic variables such as gender, age, work tenure, and education differed in experience regarding job insecurity [75] and performance [72]. Turning to current research, the Mann–Whitney U test (Table 4) showed a statistically significant difference between the job insecurity (U = 11,652.500, *p* < 0.05) and OCB (U = 11,986.000, *p* < 0.05) of males and females.

From Table 5 it is seen that the Mann–Whitney test did not reveal any statistically significant differences concerning job insecurity, task performance and OCB depending on the education level. This leads to the idea that when working as a robotised production line operator in the furniture sector, education level does not matter for feeling higher or lower job insecurity or for evaluating performance as higher or lower.

The Kruskal–Wallis test (Table 6) showed a statistically significant difference in various age groups of respondents as regards their job insecurity (H = 24.040, *p* < 0.05) and OCB (H = 12.088, *p* < 0.05). It seems that respondents born in 1965–1980 felt more insecure about their jobs (mean rank—210.82), while respondents born in 2002 and later evaluated their extra-role performance as being the most expressed (mean rank—204.71).

The Kruskal–Wallis test (Table 7) did not reveal any statistically significant differences as regards job insecurity, task performance and OCB across employees with different job tenure. This leads to the idea that the number of years spent working in a particular organisation does not influence the feeling of job insecurity or the evaluation of performance.

To test the study hypotheses, multiple regression analyses were conducted (Table 8). The results are discussed further.

H1 proposes a negative relationship between job insecurity and task performance. As it is seen from Table 8, job insecurity causes a decline in task performance (−0.215, *p* < 0.01). Thus, H1 was confirmed. The same applies regarding H2. As illustrated in Table 8, job insecurity elicits lower OCB (−0.252, *p* < 0.001). Hence, support was also found for H2.

## 5. Discussion

The paper was intended to examine the relationship between job insecurity and two dimensions of employee performance. More specifically, treating job insecurity as a hindrance stressor, the paper claims for negative association between job insecurity and task performance and OCB. In doing this, the paper echoes the call in the previous literature to focus on employee performance as one of behavioural outcomes caused and influenced by job insecurity [27]. Following the view that context matters [25,32], attention was paid to manufacturing sector. More specifically, the sample consisted of robotised production line operators working in the furniture sector characterised by specific context due to high pressure for its automation and robotisation. As predicted, the findings revealed that job insecurity served as a determinant of lower task performance and lower OCB. Further, the theoretical and practical implications of the findings are discussed.

### 5.1. Theoretical Implications

The debates whether the digital technologies substitute or create jobs have been going on intensively for several years [4]. The prevailing message proclaims that robots can do the jobs of humans more efficiently [76] and this might lead to a significant decrease in the number of human employees in the currently existing jobs [8]. Incidentally, this applies for both less-skilled occupations (e.g., plant workers) and their highly skilled counterparts [76]. Turning to furniture industry, the workers in production are under high risk of being replaced as their jobs are essentially repetitive and subject to strict algorithmisation [8]. Accordingly, employees might feel insecure about their jobs. One of the first objectives of the current paper was to measure the job insecurity of particular respondents form furniture industry. The findings demonstrated a level of job insecurity that was not particularly high (mean 2.76) allowing to suggest that workers did not perceive the threat of losing the current job in the future as high [77] regardless of seeing some examples of robotisation as they work on robotised production lines. Such findings are in line with Shin et al. [78] study, where R&D professionals employed in a South Korean manufacturing company rated job insecurity similarly (mean 2.37 on a scale from 1 to 5). Regarding differences in the perception of respondents belonging to different gender, age, education, and work tenure groups, only a few statistically significant differences were revealed (Table 4, Table 5, Table 6 and Table 7). Two aspects could be highlighted. First, the findings demonstrated that employees born in 1965–1980 had a higher job insecurity in comparison to other generations. This contradicts the findings of [75] where older workers reported lower job insecurity. Second, males reported a higher job insecurity than women. Literature review confirms that the research into gender perceptions of job insecurity is not clear and presents controversial findings [75]. Thus, the current study does not support the idea that women experience less control over their own employment futures and due to this suffer from higher job insecurity.

This paper considers both task performance and OCB as outcomes, seeing that they both represent important ways by which employees can contribute to the organisation and help it attain its goals [72]. The respondents evaluated their task performance (mean 4.11) and OCB (mean 3.81) as high indeed. Such evaluation corresponds to the findings in other studies where self-reported measures of performance were used [78]. As illustrated in Table 4, statistically significant differences (U = 11,986.000, *p* < 0.05) make it possible to underline that females (mean rank 194.21) evaluated their OCB higher than men (mean rank 163.75). It could be explained by the general tendency of women for discretionary and voluntary behaviour [20].

As job insecurity is a subjective experience [43], not all employees are equally affected and individuals may experience varying degrees of uncertainty, even if they are objectively in the same work situation [56] (all of the respondents were working as robotised production line operators). As such, job insecurity may trigger contradicting reactions. Usually, the understanding how job insecurity is related to employee performance is explained through the hindrance–challenge stressor model [27]. In the current paper, following the idea of a hindrance stressor, it was predicted that job insecurity would be an undesirable work-related demand that interferes with task accomplishment. The findings supported the mentioned assumption by revealing that job insecurity served as one of the reasons which negatively affected task performance. However, it should be admitted that occupational health psychology literature considers job insecurity as just one example among stressors and in comparison to other work-related stressors, job insecurity is one of the least important amongst the most important stressors [79]. Coming back to the current research, the results are consistent with the majority of previous studies that have explored the relationship between job insecurity and task performance. For instance, the findings are in line with findings of Schreuers et al. [20], while Shin and Hur [21] demonstrated that job insecurity was strong enough to exert a long-term negative effect on the task performance of service employees.

Turning to the relationship between job insecurity and OCB, the same view of job insecurity as a hindrance stressor was proposed. The findings supported the notion that job insecurity led to lower OCB (−0.252, *p* < 0.001). That is, when employees perceive a threatening stressor such as job insecurity, they use up energy to cope with it, diverting efforts away from performing extra-role job behaviours [27]. This notwithstanding, the current study does not support the findings of Schreuers et al. [20] where no significant negative association was observed between job insecurity and OCB. Although the latter might represent the case when employees do not increase or decrease their discretionary efforts when exploring job insecurity [20], the current research belongs to the literature stream, which argues, based on empirical evidence, that job insecurity damages OCB by diminishing employee efforts to go “above and beyond the call of duty” [53] (p. 60).

Summing up, the results of the current study support the notion that job insecurity is associated with impaired performance due to its being a stressor. Thus, the role of job insecurity as a hindrance stressor is confirmed.

### 5.2. Practical Implications

In addition to the theoretical implications, the research has some managerial implications for practitioners. Based on the finding that job insecurity impairs task performance and OCB, organisations are encouraged to design some strategies and take some actions, which are concerned with eliminating or reducing job insecurity as such. This is an extremely heavy task due to the nature of job insecurity and its inevitability in today’s organisations, especially in the manufacturing industry where the number of robots is growing. Nonetheless, several aspects that might be taken into consideration by practitioners are laid down below.

In general, job insecurity is problematic as it implies unpredictability and uncontrollability [13]. Drawing upon common agreement in the literature, negative consequences of job insecurity could be reduced while reducing the mentioned qualities, namely unpredictability and uncontrollability [13,78]. For this, several ways are proposed, such as communication [80], participation in decision making [81], and enhancement of organisational justice [82].

The first strategy refers to organisational communication seeing that the combination of fear to lose the job and lack of information may be extremely stressful for employees [80]. Clarifying and communicating managerial expectations with respect to humans and robotisation may counteract the feeling of uncertainty in employees [20]. Thus, job insecurity might be reduced through communication of future organisational plans, seeing that information is a valuable resource that increases the predictability of the working situation and makes it more understandable from the angle of employees [80]. Moreover, employees having access to information can gain more resources to cope with the adverse consequences of job insecurity [27,80]. The organisation’s leaders might think about an internal communication plan and communication means. The frequency, channels, structure, and content of the messages need to be discussed and carefully chosen in order to make the organisation’s plans comprehensible for all employees.

The second strategy refers to employee involvement in decision making, as it increases the control over the situation [13], thereby reducing job insecurity. In general, organisations that are more inclined to involve the employees usually take a long-term perspective on employment reduction [81] estimating the advantages and drawbacks of work design changes. Moreover, the opportunity to discuss organisational changes increases the feeling among employees that their needs are important for the organisation and are taken into consideration [81] when automation and robotisations decisions are discussed, chosen and implemented. Participation in decision-making may take different forms, such as individual or collective.

The third strategy concerns organisational justice. In fact, employees who perceive greater justice, will have a stronger sense that they are respected and valued by the organisations [83]. Hence, employees tend to evaluate their job existence in the future to be more controllable and predictable [84]. Accordingly, they feel less insecure.

Summing up, the core idea is that one-shot approach to reducing job insecurity is unlikely to be successful. The complex of actions with respect to open communication, involving employees in decision making and increasing the feeling of fairness of the organisational policies and procedures might create a synergic effect and reduce job insecurity as such.

### 5.3. Limitations

This research has some shortcomings that should be acknowledged when interpreting the results. The first concern is related to self-report nature of the data regarding task performance and OCB. This may have increased the risk of common method variance [73,74] and other response biases such as social desirability. Attempts were made to decrease the social desirability on measurements of task performance and OCB by guaranteeing anonymity of the results and emphasising that there would be no right or wrong answers [27]. Indeed, the respondents provided favourable ratings of their own task performance and OCB, as indicated by high means for those variables (M = 4.11 for task performance; M = 3.81 for OCB). This suggests the possibility that job performance and OCB were overrated due to social desirability and evaluation apprehension. For this reason, other rated measures of task performance and OCB are recommended.

The second concern is a linking mechanism between job insecurity and performance dimensions. In order to completely understand the relationship between job insecurity, task performance, and OCB, the underlying processes should be considered [78]. Although it was outside the scope of the paper to explore possible mediators and moderatos that can strengthen or weaken the relationship between job insecurity and performance dimensions, future research nonetheless needs to consider a linking mechanism.

The third concern relies on the dimensions of employee performance. As the current paper captured only two types of employee performance, the future research could also include counterproductive and other dimensions of performance [45].

The fourth concern deals with sample specifics. The sample consisted of robotised production line operators working in the furniture sector. For this reason, the result could not be generalised to other manufacturing industries.

The fifth concern relates to possibility to explore the generalisability of the results to other cultures and countries. Since the sample was from one country, it would be interesting to examine whether the job insecurity–performance relationship varies across countries and whether this variation depends on specific country-level characteristics. In this respect, the current research supports the interest and call that was also raised by some earlier studies [19,25,27].

The sixth concern is related to the fact that the objective predictors of job insecurity were neglected in the current research [2,75]. During survey, the data on organisations’ size or employee wages were not collected. In order to have a complete picture of job insecurity and its impact on employee performance, further research should consider previously mentioned relevant data”

## 6. Conclusions

The aim of the paper was to explore the relationship between job insecurity and task performance and OCB. Results of the survey carried out among the robotised production line operators working in the furniture sector revealed that job insecurity had a negative effect on task performance and OCB. More specifically, job insecurity causes impaired task performance by hindering the employees from exhibiting the behaviours, which include fundamental job responsibilities from the job description. Turning to OCB, job insecurity is detrimental to OCB as employees show lower prosocial behaviour, which supports the psychological and social environment in which task performance takes place. Summing up, the results are in line with the stress theory suggesting that job insecurity is associated with impaired performance due to its being a stressor, namely a hindrance stressor.

## Figures and Tables

**Table 1:** Main indicators, 2015 [62].

Main Indicators	Number
Operating companies	872
Number of employees	27,724
Production value	€1.4 bn
Added value	€391 mill
Export of goods of Lithuanian origin	€1.2 bn
Investments in tangible assets	€61.5 mill

**Table 2:** Respondents’ profile.

	%
Furniture industry	100
Working on robotised production line	100
Gender	
Male	61
Female	39
Age	
Generation Z (date of birth 2002 and later)	5
Generation Y (date of birth 1981–2001)	50
Generation X (born in 1965–1980)	35
Baby boom generation (born in 1946–1964)	10
Education	
Higher education	41
Non-degree	59
Time worked for the organisation (job tenure)	
Under 1 year	4
1–3 years	21
3–5 years	38
5–10 years	30
10 years and over	7

**Table 3:** Mean, standard deviation and Spearman correlations.

	Mean	SD	1	2	3	4	5	6
1. Gender	1.39	0.49						
2. Age	2.50	0.74	0.056					
3. Education	1.59	0.49	0.035	−0.122 *				
4. Time worked for the organisation	3.13	0.96	−0.046	−0.370 **	−0.077			
5. Job insecurity	2.76	0.65	−0.164 **	−0.152 **	0.013	0.004		
6. Task performance	4.11	0.57	0.016	0.091	−0.034	−0.094	−0.261 **	
7. OCB	3.81	0.77	0.157 **	0.139 **	0.031	−0.070	−0.249 **	0.496 **

Notes: *n* = 350; * *p* < 0.05; ** *p* < 0.01; SPSS 23 was used to calculate the means and standard deviations; reported means of latent variables were zero in cross-sectional analyses. Correlations between variables such as job insecurity were only measured with one construct (i.e., not a latent variable).

**Table 4:** Gender differences in job insecurity, task performance and OCB (Mann–Whitney test).

	Female Mean Rank	Male Mean Rank	Mann–Whitney U Test	Z	Sig.
Job insecurity	154.31	188.80	11,652.500	−3.134	0.002
Task performance	175.99	175.20	14,447.000	−0.072	0.942
OCB	194.21	163.75	11,986.000	−2.751	0.006

**Table 5:** Education differences in job insecurity, task performance and OCB (Mann–Whitney test).

	University Degree Mean Rank	Non-University Degree Mean Rank	Mann–Whitney U Test	Z	Sig.
Job insecurity	173.45	176.95	14,565.500	−0.322	0.748
Task performance	178.19	173.60	14,472.500	−0.426	0.670
OCB	171.80	178.12	14,325.500	−0.578	0.563

**Table 6:** Age differences in job insecurity, task performance and OCB (Kruskal –Wallis H test).

	Born in 1946–1964 Mean Rank	Born in 1965–1980 Mean Rank	Born in 1981–2001 Mean Rank	Born in 2002 and Later Mean Rank	Kruskal–Wallis H Test	df	Sig.
Job insecurity	162.47	210.82	153.98	168.79	24.040	3	0.000
Task performance	179.71	161.11	184.13	181.82	4.039	3	0.257
OCB	182.07	150.65	188.77	204.71	12.088	3	0.007

**Table 7:** Working years’ differences in job insecurity, task performance and OCB (Kruskal–Wallis H test).

	Less than 1 Year Mean Rank	1–3 Years Mean Rank	3–5 Years Mean Rank	5–10 Years Mean Rank	More than 10 Years Mean Rank	Kruskal–Wallis H Test	df	Sig.
Job insecurity	176.93	167.95	181.72	168.36	194.93	2.332	3	0.675
Task performance	163.37	189.10	179.92	170.00	138.78	5.340	3	0.254
OCB	188.87	188.95	169.10	183.10	126.46	8.152	3	0.086

**Table 8:** Multiple regression results.

	Dependent Variable: (Standardised β)		
Independent Variables	Job Insecurity	Job Insecurity H1	Job Insecurity H2
*Control variables*			
Gender	−0.162 *	−0.165 **	−0.124 *
Age	−0.161 *	−0.154 **	−0.133 *
Education	0.007	−0.001	0.020
Time worked for the organisation	−0.041	−0.060	−0.045
*Constructs*			
Task performance		−0.215 ***	
OCB			−0.252 ***
R2	0.052	0.097	0.112
Total F	4.686 **	7.394 ***	8.693 ***
Adjusted R2	0.041	0.084	0.099

Notes: * *p* < 0.05; ** *p* < 0.01; *** *p* < 0.001.

## Appendix A. Survey Items

Job insecurity (De Witte [70]).

(1) I feel insecure about the future of my job.
(2) I am sure I can keep my job (reverse coded).
(3) Chances are, I will soon lose my job.
(4) I think, I might lose my job in the near future.

Task performance (Goodman and Svyantek, [71]).

(1) I achieve the objectives of my job.
(2) I fulfil all the requirements of my job.
(3) I demonstrate expertise in all job-related tasks.

OCB (Verburg et al. [72]).

(1) I accept added responsibility when others are absent.
2) I help others out when I can see they have a heavy workload.
(3) I assist others with their work, even when not directly asked.
(4) My attendance at work is above the norm.
(5) I give advance notice when unable to come to work.

## References

[1] Howard A.E. (1995). The Changing Nature of Work..

[2] Sverke M., Hellgren J. (2002). The Nature of Job Insecurity: Understanding Employment Uncertainty on the Brink of a New Millennium. Appl. Psychol..

[3] Coupe T. (2019). Automation, job characteristics and job insecurity. Int. J. Manpow..

[4] Dengler K., Matthes B. (2018). The impacts of digital transformation on the labour market: Substitution potentials of occupations in Germany. Technol. Forecast. Soc. Chang..

[5] Caruso L. (2018). Digital innovation and the fourth industrial revolution: Epochal social changes?. AI Soc..

[6] Bhargava A., Bester M., Bolton L. (2020). Employees’ Perceptions of the Implementation of Robotics, Artificial Intelligence, and Automation (RAIA) on Job Satisfaction, Job Security, and Employability. J. Technol. Behav. Sci..

[7] Webster C., Ivanov S. (2020). Robotics, Artificial Intelligence, and the Evolving Nature of Work. Digital Transformation in Business and Society.

[8] Ivanov S.H. (2017). Robonomics-principles, benefits, challenges, solutions. Yearb. Varna Univ. Manag..

[9] Dekker F., Salomons A., van der Waal J. (2017). Fear of robots at work: The role of economic self-interest. Socio Econ. Rev..

[10] Nica E. (2016). Will Technological Unemployment and Workplace Automation Generate Greater Capital–Labor Income Imbalances?. Econ. Manag. Financ. Mark..

[11] Nam T. (2019). Technology usage, expected job sustainability, and perceived job insecurity. Technol. Forecast. Soc. Chang..

[12] Shoss M.K. (2017). Job Insecurity: An Integrative Review and Agenda for Future Research. J. Manag..

[13] De Witte H. (2005). Job insecurity: Review of the international literature on definitions, prevalence, antecedents and consequences. SA J. Ind. Psychol..

[14] De Witte H., Pienaar J., De Cuyper N. (2016). Review of 30 Years of Longitudinal Studies on the Association Between Job Insecurity and Health and Well-Being: Is There Causal Evidence?. Aust. Psychol..

[15] Darvishmotevali M., Ali F. (2020). Job insecurity, subjective well-being and job performance: The moderating role of psychological capital. Int. J. Hosp. Manag..

[16] László K.D., Pikhart H., Kopp M.S., Bobak M., Pajak A., Malyutina S., Salavecz G., Marmot M. (2010). Job insecurity and health: A study of 16 European countries. Soc. Sci. Med..

[17] Karatepe O.M., Rezapouraghdam H., Hassannia R. (2020). Job insecurity, work engagement and their effects on hotel employees’ non-green and nonattendance behaviors. Int. J. Hosp. Manag..

[18] Ali M., Ali I., Albort-Morant G., Leal-Rodríguez A.L. (2020). How do job insecurity and perceived well-being affect expatriate employees’ willingness to share or hide knowledge?. Int. Entrep. Manag. J..

[19] Staufenbiel T., König C.J. (2010). A model for the effects of job insecurity on performance, turnover intention, and absenteeism. J. Occup. Organ. Psychol..

[20] Schreurs B.H.J., Hetty van Emmerik I., Günter H., Germeys F. (2012). A weekly diary study on the buffering role of social support in the relationship between job insecurity and employee performance. Hum. Resour. Manag..

[21] Shin Y., Hur W.-M. (2019). When Do Service Employees Suffer More from Job Insecurity? The Moderating Role of Coworker and Customer Incivility. Int. J. Environ. Res. Public Health.

[22] Sverke M., Låstad L., Hellgren J., Richter A., Näswall K. (2019). A Meta-Analysis of Job Insecurity and Employee Performance: Testing Temporal Aspects, Rating Source, Welfare Regime, and Union Density as Moderators. Int. J. Environ. Res. Public Health.

[23] Probst T.M., Jiang L., López Bohle S.A. (2019). Job insecurity and impression management. Career Dev. Int..

[24] Becton J.B., Carr J.C., Mossholder K.W., Walker H.J. (2017). Differential Effects of Task Performance, Organizational Citizenship Behavior, and Job Complexity on Voluntary Turnover. J. Bus. Psychol..

[25] Gilboa S., Shirom A., Fried Y., Cooper C. (2008). A meta-analysis of work demand stressors and job performance: Examining main and moderating effects. Pers. Psychol..

[26] Cheng G.H.L., Chan D.K.S. (2008). Who Suffers More from Job Insecurity? A Meta-Analytic Review. Appl. Psychol..

[27] Piccoli B., Reisel W.D., De Witte H. (2019). Understanding the Relationship Between Job Insecurity and Performance: Hindrance or Challenge Effect?. J. Career Dev..

[28] Bowen H.R., Mangum G.L. (1966). Technology and the American Economy.

[29] Schwab K. (2017). The Fourth Industrial Revolution.

[30] Moniz A., Krings B.-J. (2016). Robots Working with Humans or Humans Working with Robots? Searching for Social Dimensions in New Human-Robot Interaction in Industry. Societies.

[31] Zhang P. (2019). Automation, wage inequality and implications of a robot tax. Int. Rev. Econ. Financ..

[32] Rafiq M., Chin T. (2019). Three-Way Interaction Effect of Job Insecurity, Job Embeddedness and Career Stage on Life Satisfaction in A Digital Era. Int. J. Environ. Res. Public Health.

[33] Autor D.H. (2015). Why Are There Still So Many Jobs? The History and Future of Workplace Automation. J. Econ. Perspect..

[34] Graetz G., Michaels G. (2018). Robots at Work. Rev. Econ. Stat..

[35] World Economic Forum (2018). The Future of Jobs Report.

[36] Brynjolfsson E., McAfee A. (2014). The Second Machine Age: Work, Progress, and Prosperity in a Time of Brilliant Technologies.

[37] Villani V., Pini F., Leali F., Secchi C. (2018). Survey on human–robot collaboration in industrial settings: Safety, intuitive interfaces and applications. Mechatronics.

[38] Acemoglu D., Restrepo P. (2020). Robots and Jobs: Evidence from US Labor Markets. J. Polit. Econ..

[39] Acemoglu D., Autor D. (2011). Skills, Tasks and Technologies: Implications for Employment and Earnings. Handbook of Labor Economics.

[40] Greenhalgh L., Rosenblatt Z. (1984). Job Insecurity: Toward Conceptual Clarity. Acad. Manag. Rev..

[41] Brondino M., Bazzoli A., Vander Elst T., De Witte H., Pasini M. (2020). Validation and measurement invariance of the multidimensional qualitative job insecurity scale. Qual. Quant..

[42] Jiang L., Lavaysse L.M. (2018). Cognitive and Affective Job Insecurity: A Meta-Analysis and a Primary Study. J. Manag..

[43] Vander Elst T., De Witte H., De Cuyper N. (2014). The Job Insecurity Scale: A psychometric evaluation across five European countries. Eur. J. Work Organ. Psychol..

[44] Viswesvaran C., Ones D.S. (2000). Perspectives on Models of Job Performance. Int. J. Sel. Assess..

[45] Pradhan R.K., Jena L.K. (2017). Employee Performance at Workplace: Conceptual Model and Empirical Validation. Bus. Perspect. Res..

[46] Borman W.C., Motowidlo S.M. (1993). Expanding the Criterion Domain to Include Elements of Contextual Performance.

[47] Borman W.C., Motowidlo S.J. (1997). Task Performance and Contextual Performance: The Meaning for Personnel Selection Research. Hum. Perform..

[48] Van Scotter J.R. (2000). Relationships of Task Performance and Contextual Performance with Turnover, Job Satisfaction, and Affective Commitment. Hum. Resour. Manag. Rev..

[49] Conway J.M. (1999). Distinguishing contextual performance from task performance for managerial jobs. J. Appl. Psychol..

[50] Rotundo M., Sackett P.R. (2002). The relative importance of task, citizenship, and counterproductive performance to global ratings of job performance: A policy-capturing approach. J. Appl. Psychol..

[51] Organ D.W. (1988). Organizational Citizenship Behavior: The Good Soldier Syndrome.

[52] Organ D.W. (1997). Organizational Citizenship Behavior: It’s Construct Clean-Up Time. Hum. Perform..

[53] Bolino M.C., Turnley W.H. (2003). Going the extra mile: Cultivating and managing employee citizenship behavior. Acad. Manag. Perspect..

[54] Rousseau D.M. (1995). Psychological Contract: Understanding of Written and Unwritten Agreements.

[55] Selenko E., Mäkikangas A., Stride C.B. (2017). Does job insecurity threaten who you are? Introducing a social identity perspective to explain well-being and performance consequences of job insecurity. J. Organ. Behav..

[56] Lepine J.A., Podsakoff N.P., Lepine M.A. (2005). A Meta-Analytic Test of the Challenge Stressor–Hindrance Stressor Framework: An Explanation for Inconsistent Relationships Among Stressors and Performance. Acad. Manag. J..

[57] Cavanaugh M.A., Boswell W.R., Roehling M.V., Boudreau J.W. (2000). An empirical examination of self-reported work stress among U.S. managers. J. Appl. Psychol..

[58] Roll L.C., Siu O., Li S.Y.W. (2015). The job insecurity-performance relationship in Germany and China: The buffering effect of uncertainty avoidance. Psihol. Resur. Um..

[59] Probst T.M., Stewart S.M., Gruys M.L., Tierney B.W. (2007). Productivity, counterproductive and creativity: The ups and downs of job insecurity. J. Occup. Organ. Psychol..

[60] Lukšytė V., Melnikas B. Baldų gamyba ir eksporto plėtra ekonomikos globalizacijos sąlygomis. Proceedings of the 21st Conference “Business in XXI Century”.

[61] Lietuvos Statistikos Departamentas Statistinių Rodiklių Analizė. https://osp.stat.gov.lt/statistiniu-rodikliu-analize#/.

[62] Versli Lietuva (2017). Lietuvos Baldų Gamybos Pramonė.

[63] Han Y. (2020). Furniture Report 2020.

[64] Rutkauskaitė R. SBA žengia į robotų diegimo rinką—įsigijo bendrovę “Robotex”. https://www.vz.lt/pramone/2019/06/17/sba-zengia-i-robotu-diegimo-rinka--isigijo-bendrove-robotex#ixzz6ec4pOn9r.

[65] Markevičienė E. Pramonei „lean“ nebeužteks, reikia technologijų. https://www.vz.lt/pramone/2018/06/13/pramonei-lean-nebeuzteks-reikia-technologiju#ixzz5INvjRRAh.

[66] Statybunaujienos. Lt Pramonės Revoliucija ir Robotai Lietuvos Įmonėse: Skelbia Geriausio Sprendimo Paieškas. http://www.statybunaujienos.lt/naujiena/Pramones-revoliucija-ir-robotai-Lietuvos-imonese-skelbia-geriausio-sprendimo-paieskas/12488.

[67] OECD (2018). Putting Faces to the Jobs at Risk of Automation.

[68] Liuima J. (2017). Kurios Lietuvos Pramonės ir Paslaugų Sritys Bus Svarbiausios 2025 Metais?.

[69] Lietuvos pramonininkų konfederacija (2019). Lietuvos Pramonės Lūkesčių Indeksas. 2019 Metų i Pusmetis.

[70] De Witte H., Bouwen R., de Witte K., de Witte H., Taillieu T. (2000). Arbeidsethos en jobonzekerheid: Meting en gevolgen voor welzijn, tevredenheid en inzet op het werk (Work ethic and job insecurity: Assessment and consequences for well-being, satisfaction and performance at work). Van Groep Naar Gemeenschap (From Group to Community).

[71] Goodman S.A., Svyantek D.J. (1999). Person–Organization Fit and Contextual Performance: Do Shared Values Matter. J. Vocat. Behav..

[72] Verburg R.M., Nienaber A.-M., Searle R.H., Weibel A., Den Hartog D.N., Rupp D.E. (2018). The Role of Organizational Control Systems in Employees’ Organizational Trust and Performance Outcomes. Gr. Organ. Manag..

[73] Podsakoff P.M., MacKenzie S.B., Lee J.-Y., Podsakoff N.P. (2003). Common method biases in behavioral research: A critical review of the literature and recommended remedies. J. Appl. Psychol..

[74] Podsakoff P.M., MacKenzie S.B., Podsakoff N.P. (2012). Sources of Method Bias in Social Science Research and Recommendations on How to Control It. Annu. Rev. Psychol..

[75] Keim A.C., Landis R.S., Pierce C.A., Earnest D.R. (2014). Why do employees worry about their jobs? A meta-analytic review of predictors of job insecurity. J. Occup. Health Psychol..

[76] Lee C., Huang G.-H., Ashford S.J. (2018). Job Insecurity and the Changing Workplace: Recent Developments and the Future Trends in Job Insecurity Research. Annu. Rev. Organ. Psychol. Organ. Behav..

[77] Vander Elst T., De Cuyper N., Baillien E., Niesen W., De Witte H. (2016). Perceived Control and Psychological Contract Breach as Explanations of the Relationships Between Job Insecurity, Job Strain and Coping Reactions: Towards a Theoretical Integration. Stress Health.

[78] Shin Y., Hur W.M., Moon T.W., Lee S. (2019). A motivational perspective on job insecurity: Relationships between job insecurity, intrinsic motivation, and performance and behavioral outcomes. Int. J. Environ. Res. Public Health.

[79] De Witte H. (1999). Job Insecurity and Psychological Well-being: Review of the Literature and Exploration of Some Unresolved Issues. Eur. J. Work Organ. Psychol..

[80] Jiang L., Probst T.M. (2014). Organizational communication: A buffer in times of job insecurity?. Econ. Ind. Democr..

[81] Gallie D., Felstead A., Green F., Inanc H. (2017). The hidden face of job insecurity. Work. Employ. Soc..

[82] Greenberg J. (1990). Organizational Justice: Yesterday, Today, and Tomorrow. J. Manag..

[83] Cropanzano R., Rupp D.E., Mohler C.J., Schminke M. (2001). Three Roads to Organizational Justice.

[84] Loi R., Lam L.W., Chan K.W. (2012). Coping with Job Insecurity: The Role of Procedural Justice, Ethical Leadership and Power Distance Orientation. J. Bus. Ethics.

